# Mr. Lyall, on Scarification

**Published:** 1809-11

**Authors:** Robert Lyall

**Affiliations:** Infirmary, Manchester


### *To the Editors of the Medical and Phyſical Journal.*

Gentlemen,

Having observed in the last Number of your Journal, a request by a "Country Practitioner, that some of your Readers would detail the operation of Scarification with minuteness, and explain on what circumstances the success of it depends," I am induced to subjoin a few observations on that subject, which may perhaps be of advantage to him, if they are thought worthy of a place in your excellent Miscellany.

Before entering upon the subject of consideration, it may be necessary to premise, that scarification is an operation which I have very often performed. The scarficator here used, seems to be the same as that which the Country Practitioner speaks off, and the cupping glasses are also of the same shape as those which he mentions; but we have them of various diameters at the mouth, from 1 ¼ to

1¼ to 2½ inches, so as to suit the part accurately to which it is intended they are to be applied.

To succeed in the first part (cutting part) of this operation. 1st. It is necessary that the scarificator have a good spring, and be otherwise in good order; and, 2dly, That too much depth be not given to the lancet before the instrument is applied. That the state of the spring may be examined, it will be necessary to try the instrument, and to regulate the depth of the lancets; it (the scarificator) must be half cocked, and then by turning the screw, we may expose any length (or what I mean above, by depth) of the lancets we please. It is a very common idea that the more length you allow the lancets, the greater will be the success. When I first began to use the scarificator, I also entertained this notion, and commonly turned the screw until it would turn no more, thus exposing as much of the lancets as I could; but experience soon taught me that this was not the way to succeed. 1st Because, if so much of the lancets could be exposed (trying previous to the operation) when the instrument was applied, I found that instead of the lancets cutting, "the box in which they were contained was elevated by them as by leavers," unless very great pressure was used, greater than I myself generally could apply. 2d. Because, if sufficient pressure was used to prevent the box from being elevated, "the lancets were arrested in their progress," and I was obliged to pull them round (through) by means of the trigger, owing to want of strength in the spring; or if this was not done, the lancets did not cut sufficiently, and of course a free discharge of blood was not procured. These then were the accidents which occurred to me, till I began to consider that the spring was only calculated to overcome a certain resistance, and that if I would regulate the depth of the lancets, antecedent to the application of the instrument (considering also the part to which it was to be applied, the hardness or softness of the integuments of the parts, &c.) so as that the spring by its own force would be able to bring them completely round, when it was applied to the part to be scarified, I would then certainly avoid these disagreeable occurrences.

Since that time, before applying the scarificator, I have generally turned the screw, the instrument being half-cocked, until about ⅔ of the length of the lancets could be exposed; and since attending to this circumstance, my success has been much greater than formerly. No doubt, however, the depth given to the lancets (previous to the

 operation)

operation) must be regulated by the strength of the spring, and the texture of the parts which are intended to be cut; circumstances which will soon be determined, if the operator is in the frequent habit of using this instrument.

From the manner in which the "Country Practitioner" mentions the case in which he only procured one or two ounces of blood, I have little doubt but inattention to, or want of knowledge of these circumstances, was the cause of his (as well as many others) failure.

So much then for the scarificator, and the manner of using it. We must next speak of the cupping glasses. The Country Practitioner asks, "If the form and size of the glass" is of "any particular importance?" To which I reply, that I cannot conceive there can be any material difference, of whatever form or size the glass be, providing that as compleat a vacuum is procured as is in general necessary. I have succeeded as well with a wine glass as with a cupping glass, but am ready to acknowledge, that the latter is by much the most convenient. The wine glass may be used when a cupping glass cannot be procured. The best manner (and which is, I believe, the manner used by particular persons in London, who perform this operation with dexterity) of applying the cupping glasses, as far as I know, is the following. 1st. The cup should be put into warm water while the patient is preparing; having near at hand a lighted candle, a piece of paper rolled into a cylindrical form, and a towel, the scarificator should be applied. The cupping glass should then be quickly dried, and the paper being lighted, should be held horizontally over, and pretty near the scarified part; the cupping glass is then to be so placed as to receive the flame from the paper into its centre, and when the air is thought to be pretty much rarefied, the lighted paper should be withdrawn quickly, and the cupping glass instantaneously placed over the orifices. These are the most essential points then to be observed in scarification; but there are others to be considered, which I think have already been ably pointed out by you (the Editors). By attending then to the circumstances above mentioned, in general, we shall be able to take as much blood as we would wish; but yet there are some instances, in which, notwithstanding every precaution, we shall fail. Whenever I feel the skin, I can generally tell whether I am to expect much blood or not. If the integuments are soft and thin I look forwards for a good discharge; but if indurated or puffy, for the contrary. In some cases, when the skin has has been thick or puffy, after applying the scarificator, and finding little blood discharged, I have taken a common lancet and made the openings a little deeper, which has been followed by the wished for effect. I hope the Country Practitioner will now be satisfied with this answer, made to his request; but if not, I shall be glad to give him any other information in my power. I trust, at least, he will attend to the subject, and inform us whether he has been more successful.

As you expressed a wish also that this subject should be noticed, I have little doubt but that these observations will meet with that attention which you think them deserving of.

I am, &c.

ROBERT LYALL.

*Infirmary, Manchester,*
*Sept.* 6, 1809.

P. S. Another of your Correspondents has made a proposal concerning the fitting of the beak of the gorget into the groove of the staff, its edges being approximated, &c; at the same time he justly conjectures, that the same contrivance may have been before proposed. Without making any remarks on this subject, I have merely to inform him, that the present celebrated Professor Monro, sen. has for many years past, shown in the anatomical theatre an instrument of the same kind, which I had an opportunity of seeing about a year and a half ago.

